# Inward Rectifier Potassium (Kir) Channel Inhibitors Protect Citrus from the Asian Citrus Psyllid by Inducing Toxicity and Inhibition of Feeding

**DOI:** 10.1021/acs.jafc.5c17254

**Published:** 2026-03-24

**Authors:** Flinn O’Hara, Alexandra Cremades, Na Xie, Erik L. Roldán, Miltan Chandra Roy, Sandipa Gautam, Mamoudou Setamou, Amar Chittiboyina, Troy D. Anderson, Lukasz L. Stelinski, Daniel R. Swale

**Affiliations:** † Emerging Pathogens Institute, Department of Entomology and Nematology, University of Florida, Gainesville, Florida 32610, United States; ‡ Citrus Research and Education Center, Department of Entomology and Nematology, 3463 University of Florida, Lake Alfred, Florida 33850, United States; § University of California Agricultural and Natural Resources, Statewide IPM Program, Exeter, California 93221, United States; ∥ Citrus Center, Texas A&M University Kingsville, Weslaco, Texas 78599, United States; ⊥ National Center for Natural Products Research, School of Pharmacy, 8083 University of Mississippi, University, Mississippi 38677, United States; # Department of Entomology, University of Nebraska, Lincoln, Nebraska 68583, United States

**Keywords:** citrus greening disease, kir channels, antifeedant, insecticide, C. Las acquisition, Huanglongbing

## Abstract

Huanglongbing (HLB), caused by the phloem-restricted bacterium *Candidatus Liberibacter asiaticus* (*C. Las*), is vectored by the Asian citrus psyllid (ACP) and threatens global citrus production. We demonstrate that VU041 (C_19_H_20_F_3_N_3_O), a small-molecule inhibitor of inward rectifier potassium (Kir) channels, provides a dual-mechanism approach to management of Huanglongbing (HLB) disease by combining acute toxicity with antifeedant activity. Topical and tarsal contact bioassays revealed that VU041 induced rapid mortality to ACP (24 h LD_50_ = 25 ng/insect) and reduced salivary gland secretion to suppress feeding behaviors. In semifield acquisition assays on *C. Las*-infected citrus, foliar application of VU041 significantly reduced both ACP survival (up to 96% at 365 ppb) and *C. Las* acquisition in surviving ACP. Combined, these results indicate that VU041 represents a promising chemical lead for developing insecticides that simultaneously kill ACP vectors and block pathogen acquisition at sublethal concentrations, representing a potentially transformative approach to reducing HLB-mediated losses in citrus production.

## Graphical abstract

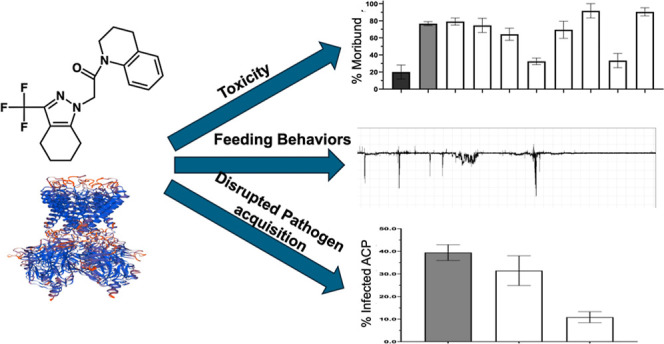

## Introduction

The citrus industry is a significant component of the U.S. economy, with an annual economic impact averaging approximately $9–10 billion USD in Florida (FL) and ∼7.5 billion USD in California (CA) between 2019 and 21. Infestation of citrus grooves with Asian citrus psyllid (ACP; *Diaphorina citri*) has devastated citrus production because the insect transmits *Candidatus Liberibacter asiaticus*’ (*C. Las*), the bacterium that causes Huanglongbing disease (HLB).^1,2^ This bacterium is transmitted by *D. citri* in a persistent, propagative manner during feeding on phloem sap.^3,4^ ACP infestations and transmission of *C. Las* threatens all citrus growing regions in the United States, including FL, Texas (TX), and CA, which will continue to exacerbate the economic burden of this insect pest within the US.^5^ HLB is not restricted to the US and is recognized as global problem affecting citrus with an all-time high HLB disease incidence (38%) being reported in Brazil in 2023.^6^ The disease leads to rapid tree decline, triggers erratic and early flowering, increases fruit drop, and results in distorted, poor-quality fruit with a bitter taste.^7,8^ Despite advancements in plant breeding^9,10^ and application of antibiotics against the pathogen,^11–13^ no sustainable solution for preventing or eliminating HLB in citrus grooves has been commercialized. The absence of viable strategies to prevent HLB, along with reduced access to effective insecticides for ACP control because of evolved resistance to existing chemistries,^14^ highlights the urgent need to develop novel insecticide technologies targeting ACP feeding biology and survival to prevent the spread of HLB.^15^

Recent work has highlighted the potential of organically approved and naturally derived insecticides for ACP management, including plant-based and mineral formulations such as pyrethrins, azadirachtin-based products, spinosyns, soaps, and diatomaceous materials, which can suppress ACP populations but often provide inconsistent or short-lived control under field conditions.^16^ In parallel, renewable bioproducts derived from lignocellulosic biomass, including sucrose fatty acid esters and phenolic compounds, have emerged as promising eco-friendly alternatives, with laboratory and greenhouse studies demonstrating measurable contact toxicity to ACP adults while offering favorable environmental and regulatory profiles.^17^ These studies underscore both the promise and the limitations of current ‘green’ chemistries, reinforcing the need for novel compounds that combine environmental compatibility with improved potency, residual performance, and functional impacts on feeding behavior.

Accordingly, this study aimed to establish a dual-mechanism approach for HLB mitigation by identifying chemical insecticides and an ACP target essential for psyllid survival and feeding. Psyllid feeding inhibitors may improve tree health and limit HLB spread by either killing the vector or, at sublethal doses, disrupting the feeding behavior through which the pathogen is transmitted. The salivary gland is an attractive target for reducing arthropod-vectored pathogens because it is essential for successful feeding by Hemiptera and other pathogen-transmitting insects.^18,19^ Hemipteran salivary glands produce two types of saliva: a “gel” saliva that forms a protective sheath and enables stable phloem feeding, and a “watery” saliva that delivers effectors capable of suppressing plant immune responses.^20^ Disease severity often correlates with the volume of saliva injected during feeding, as many pathogens require a threshold infectious dose; thus, reducing ACP salivation is expected to diminish HLB severity.^21^

*C. Las* is phloem-restricted^22^ and should not be acquired by uninfected ACP adults exposed to *C. Las*-infected plants if feeding is interrupted prior to phloem ingestion.^23^ ACP salivation and feeding also suppress plant immunity and impede tree growth, which likely contributes to the observed yield benefits in grooves where vector suppression is maintained even after 100% of trees express HLB symptoms. These findings demonstrate the potential for salivary gland-targeted antifeedants to improve citrus health through two complementary mechanisms: (1) blocking ACP feeding to interrupt *C. Las* acquisition and transmission, and (2) reducing exposure of trees to psyllid salivary proteins that disrupt plant physiology. Despite the clear advantages of inhibiting ACP salivation and feeding, progress has been limited by gaps in identifying druggable salivary gland targets and suitable chemical scaffolds, hindering the development of ACP-directed antifeedants.

Inward rectifier potassium (Kir) channels conduct K^+^ currents into cells at hyperpolarizing membrane potentials more readily than out of cells at depolarizing membrane potentials and have been found to play critical roles in multiple mammalian^24,25^ and arthropod^24,26–31^ physiological systems.^32^ Kir channels have been shown to be critical for insect central nervous system function^26,33,34^ and pharmacological inhibition as well as gene knockdown of salivary gland Kir channels leads to reduced saliva, altered protein secretion, impaired feeding, and increased mortality across multiple arthropod species.^18,19,35–38^ Considering (1) the critical role of Kir channels in insect neural systems, (2) the importance of salivary secretions for ACP feeding and survivorship, (3) that *C. Las* is a phloem restricted bacterial pathogen whose acquisition and transmission require ACP phloem feeding, (4) the detrimental effects of ACP salivary secretions on citrus tree health,^5,15^ and (5) the reported ability of VU041 (C_19_H_20_F_3_N_3_O) to inhibit phloem feeding in aphids,^18,38^ we hypothesized that pharmacological inhibition of Kir channels would reduce salivary secretions and phloem feeding in ACP leading to death via starvation. Therefore, this study integrated novel pharmacological, electrophysiological, and biological assays to describe the role of Kir channels in ACP salivary gland function, feeding efficiency, and *C. Las* acquisition. Our current results inform future development of novel antifeedant insecticides, RNAi-based tools, or transgenic citrus varieties aimed at reducing economic losses caused by HLB in citrus.

## Materials and Methods

### Compounds and Reagents

The Kir channel inhibitor VU041 was originally identified through high-throughput screening (HTS) against the *Anopheles gambiae* Kir1 channel.^39^ VU041 used in this study was obtained via custom synthesis from Molport (Riga, Latvia), purified by column chromatography, and confirmed to be >95% pure by ^1^H NMR analysis. Dimethyl sulfoxide (DMSO) and absolute ethanol were purchased from Sigma-Aldrich (St. Louis, MO, USA). Molecular sieve OP type 3Å (Sigma-Aldrich, St. Louis, MO, USA) was added to DMSO stocks to prevent moisture absorption; 50∼2 mm beads, each with a 3 Å pore size and ≥15% water-absorbing capacity, were added per 100 mL of stock solution. Rhodamine B was also obtained from Sigma-Aldrich (St. Louis, MO, USA).

### Insect Rearing Conditions and Plant Husbandry

ACP adults were sourced from *C. Las*-free insect colonies originally established on *Citrus sinensis* L. Osbeck cv Valencia, *Murraya paniculata*, and *Bergera koenigii* at the Citrus Research and Education Center (CREC) in Lake Alfred, FL. The greenhouse was maintained at 26 ± 2 °C, 60–65% RH, and a 16:8 h (light/dark) photoperiod with a maximum photosynthetic radiation of 215 μ mol s^–1^ m^–2^. Plant husbandry was performed as previously described by our group.^40^ All experiments were performed using two year old *Citrus sinensis* L. Osbeck cv Valencia grafted onto US-812 rootstock.^41^ To ensure the colonies remained *C. Las*-free, both the psyllids and nursery-sourced *C. sinensis* plants were tested every 2–3 months using quantitative polymerase chain reaction (qPCR). *C. Las*-positive plants, confirmed by qPCR, were maintained separately after graft inoculation with infected budwood. All insect and plant cultures were maintained without insecticide exposure.

### Toxicity Bioassays of VU041 to ACP

#### Topical Toxicity Bioassays

To quantify the intrinsic insecticidal potency of VU041 against ACP, we conducted topical and tarsal-contact bioassays to generate concentration–response relationships and determine lethal dose metrics. For topical toxicity assays, we followed the method of Pridgeon et al.^42^ and Tang et al.^43^ -with minor modifications. Briefly, ACP were chilled on ice for 20 min, after which 200 nL of VU041 dissolved into absolute ethanol was applied to the abdomen using a hand-held microapplicator (Hamilton Co., Reno, NV, USA). Control treatment groups were exposed to absolute ethanol only. Treated ACP were then placed into 50 mL Falcon tubes that had been wiped with unscented dryer sheets to minimize static during toxicity assessment. For concentration–response curves and LD_50_ determinations, five doses were tested, with 10 ACP per dose, and each bioassay was replicated 2–3 times using different psyllid cohorts. Tween-80 (P1754, Sigma-Aldrich) at 0.01% was included in all working solutions of VU041 for toxicity bioassays and 0.01% Tween-80 only in absolute ethanol was used in the negative control.

#### Structure–Activity Trends Identified via Single-Dose Leaf-Dip Bioassay

To identify structural features associated with biological activity, VU041 analogs were evaluated in a single-dose leaf-dip bioassay to compare relative contact toxicity and establish preliminary structure–activity relationships. VU041 powder was dissolved in DMSO to prepare a 100 mM stock solution, which was then diluted to 1 mM using 0.01% Tween-80 in water. Working concentrations of VU041 or other chemical inhibitors were prepared by further dilution in 0.01% Tween-80 in water. A 400 μL droplet of each VU041 solution was applied to the abaxial (dorsal) surface of Valencia SPB-1–14–19 citrus leaves and allowed to dry for 24 h at 25 °C prior to initiating the assay. Tape was applied around the treated region to restrict ACP feeding exclusively to the area exposed to the compound. To confine insects to the treated region while permitting airflow, *Drosophila* culture vials (27 mm diameter × 94 mm height) were modified by drilling a hole at 40 mm and covering it with mesh. Each vial was sealed onto the treated leaf surface using clay. Five replicates of ten ACP were starved for 2 h before being introduced into each cage. Mortality rate and behavioral effects were recorded at 24-, 48-, and 72- h after initial insect exposure to treatments. Negative control treatments all contained 0.01% Tween-80 and 0.1% DMSO in water.

To provide insights into structure–activity relationships, eight structural analogs of VU041 were purchased from Molport and screened for contact toxicity via leaf dip bioassays (Figure 1A). Each compound was tested at 10 ng/cm^2^ and toxicity was assessed at 24, 48, and 72 h (Figure 1B–D).

#### Statistics for Toxicity Assessments

Adult mortality was recorded 24 h after treatment and LC_50_/LD_50_ values were estimated using nonlinear regression curve fitting with GraphPad Prism (GraphPad Software, San Diego, CA). For all assays, control mortality remained below 10% and was corrected using Abbot’s formula.^44^

### Artificial Host Feeding Assay for Salivary Sheath Secretion

An artificial diet assay was used to determine whether oral exposure to VU041 alters salivary gland secretory function in ACP, with salivary sheath length serving as a quantitative proxy for gel saliva production during feeding. The feeding chamber was adapted from previously described designs for aphids^18,45^ with modifications to create a 9.5 mm diameter chamber and to insert a cotton plug, providing approximately 5 mm of headspace. Kir channel modulators were dissolved in DMSO and then diluted into the 15% sucrose solution containing Rhodamine B dye to final concentrations. Final DMSO levels did not exceed 0.1% and all control diets contained 0.1% DMSO. Rhodamine B was incorporated at 200 ppm, and one drop of green dye (McCormick & Co., Inc. Hunt Valley, MD, USA) was added to ensure feeding by ACP and enhance salivary sheath visibility.^46^ Eight μL of the artificial diet were pipetted onto the first later of Parafilm (Bemis, Wisconsin, USA) stretched across the chamber to a thickness of approximately 15–20 μm, and a second Parafilm layer was placed over the top to prevent evaporation. Five adult psyllids were starved for 4–5 h before being introduced into each feeding chamber. After 24 h, psyllids were removed, and the Parafilm membranes were collected for measurements of salivary sheaths.

### Salivary Sheath Measurements

After 24 h of feeding, the first Parafilm layer was removed and laid flat on a microscope plate. The area directly contacted by ACP was scanned and imaged using an Eclipse FN1 microscope (Nikon) equipped with a Fusion C14440 digital camera (ORCA) and an Achromat 10× objective. Images were captured using NIS-elements acquisition software. Salivary sheaths were measured with in ImageJ software^47^ with the Fiji plugin^48^ using the segmented line tool. For each treatment group, sheaths were averaged across 25 individual ACP.

### Electrical Penetration Graph Recordings

VU041 powder was dissolved DMSO to create a 100 mM stock solution, which was then diluted to 1 mM in 0.01% Tween-80. Working concentrations were prepared by further dilution in 0.01% Tween-80 in water. VU041 + Tween-80 solution was applied to the lamina on the adaxial surface of Valencia SPB-1–14–19 citrus leaves following previously described methods.^18^ Concentrations used for EPG assays were 1 ng/cm^2^ and 10 ng/cm^2^ of VU041. Solutions were allowed to dry for 24 h at 25 °C before initiating feeding behavior assays. The treated leaf area was bordered with tape to restrict ACP feeding to the compound-exposed region. Twenty-four ACP were used per treatment group and were starved for 2 h prior to their introduction into the feeding assay. Negative control treatments included 0.01% Tween-80 and 0.1% DMSO in water.

EPG methods for analyzing psyllid probing and feeding behaviors followed our previously described methods.^18,38,49,50^ Adult ACP aged 1 to 3 days were removed from colony plants and starved for 2 h before use. A 3 cm length of 18 μm gold wire (EPG Systems, Wageningen, The Netherlands) was attached to the dorsum of each psyllid using water-based silver glue, and a reference electrode was inserted into the soil.^18^ Four psyllids were monitored simultaneously, with one psyllid per test plant, and all recordings were performed in a Faraday cage. Signals were recorded with a Giga8 DC amplifier (EPG Systems, Wageningen, The Netherlands) and an A/D conversion rate of 100 Hz. Analog signals were digitized through the integrated Giga-8d analog-digital converter and visualized and analyzed using Stylet + analysis software (EPG Systems, Wageningen, The Netherlands).

EPG signals were identified and classified according to established nomenclature used in our previous studies^18,38,49–52^ and by others.^50,53–55^ Waveforms were categorized as A–G following standard descriptions.^56,57^ Initial stylet contact with the leaf epidermis corresponds to waveform A, while initial salivation into epidermal or mesophyll tissues corresponds to waveform B.^57^ Stylet pathway activities through plant tissues produce waveform C, and first contact with phloem tissue generates waveform D.^50^ Sieve-element feeding (waveform E) consists of two subphases: secretion of watery saliva (E1) followed by passive ingestion of phloem sap (E2).^58^ Notably, E2 is always preceded by E1, indicating that the salivation into sieve elements (E1) is required for subsequent phloem ingestion (E2).^58^ Waveform F indicates derailed stylet mechanics, and waveform G represents active ingestion of xylem sap.^57^ The following feeding behaviors were quantified: (1) the proportion of psyllids reaching xylem; (2) total time spent feeding from xylem; (3) the proportion of psyllids reaching and salivating in phloem (D-waveform + E-1); (4) the proportion of psyllids ingesting phloem sap; (5) the time required for a psyllid to reach the phloem (time to first D-waveform); and (6) total time spent within phloem sieve-elements (D, E-1, E2).

### Assessing the Effect of Orally Delivered Kir dsRNA on ACP Feeding

An artificial diet consisting of a 25% (w/v) sucrose solution in sterile water containing 0.1% green food dyes (McCormick & Co., Inc. Hunt Valley, MD, USA) was prepared as a vehicle solution for dsRNA. To assess how ACP feeding was altered following knockdown of all Kir genes, a mix of Kir1 (LOC103509674), Kir2A (LOC103509672), and Kir2B (LOC103514191) dsRNA was incorporated into an artificial diet to achieve a final concentration of 100 ng/μL for each gene. This mixture is hereafter referred to as Kir dsRNA. GFP dsRNA at the same concentration served as the negative control. All dsRNA solutions were filtered through a 25 mm syringe filter (PES 0.2 μm, Fisher Scientific, Waltham, MA, USA) prior to use.

To evaluate potential effects of dsRNA on ACP adult feeding, ten 1–3 day old, *C. Las*-negative adults were membrane-fed with either Kir dsRNA-containing solution or GFP dsRNA-containing solution. For the artificial host salivary sheath length assessment, each treatment included three biological replicates (*n* = 30 ACP per treatment). For the EPG recording study, each treatment consisted of at least six biological replicates (*n* > 24 ACP per treatment).

Briefly, adults were placed into clear plastic vials (2.5 × 5.0 cm; Thornton Plastics, UT, USA) sealed with a thin layer of Parafilm and vented with a small hole (0.5 cm) at the bottom. A total of 80 μL of the treatment solution was placed onto the Parafilm, and a second layer was stretched over the top of the vial to form a sachet allowing uniform diet distribution. Vials were maintained under controlled conditions at 25 ± 2 °C, 65 ± 5% RH, and a 14:10 h (light/dark) photoperiod in a greenhouse for 120 h. The dsRNA solutions were replaced every 24 h. After 120 h of feeding, the lower Parafilm membranes were also collected to measure the salivary sheath length, as described above, to assess whether Kir knockdown altered salivary sheath formation during the artificial host dsRNA feeding assay.

In addition, four adult ACP per biological replicate (*n* ≥ 24 per treatment) were collected for EPG feeding assays. To prepare the 120 h-dsRNA-fed ACP for the EPG recording, psyllids were starved for 2 h. Psyllids were then placed on untreated, Valencia SPB-1–14–19 citrus leaves for a 24 h EPG recording period. Following recording, individuals were removed from the gold wire, transferred to labeled microcentrifuge tubes, and immediately stored at −80 °C for subsequent quantification of Kir gene knockdown in insects used for feeding behavior assays.

### Relative Quantification of ACP Kir Gene Knockdown by Digital PCR

Each adult psyllid used for the EPG study was subsequently collected to evaluate dsRNA-mediated knockdown efficiency of Kir genes using the QIAcuity Four Digital PCR System (Qiagen, Valencia, CA, USA). Total RNA was extracted from individual psyllids using TRIzol reagent (Zymo Research, Tustin, CA, USA). Briefly, each insect was homogenized in 250 μL of TRIzol using a Pellet Pestle Cordless Motor (ThermoFisher Scientific Inc., Lafayette, CO). After a 5 min incubation at room temperature, 50 μL of chloroform (ThermoFisher Scientific Inc.) was added; tubes were inverted 10 times, incubated for 2–3 min, and centrifuged at 16,000*g* for 15 min at 4 °C. The upper aqueous phase was carefully transferred to a fresh tube, mixed with an equal volume of 100% ethanol (ThermoFisher Scientific Inc.), and purified using the RNA Clean & Concentrator-5 kit (Zymo Research), including in-column DNase I treatment according to the manufacturer’s instructions. Purified total RNA was quantified using a DeNovix DS-11 FX spectrophotometer (DeNovix Inc., Wilmington, DE, USA).

For digital PCR, reactions were prepared using the QIAcuity OneStep Advanced Probe Kit (Qiagen) and loaded into QIAcuity Nanoplate 8.5k 24-well plates (Qiagen). Each reaction had a final volume of 12 μL and contained 2 μL of total RNA diluted 1:100 prior to setup, along with gene-specific primers and probes (Supporting Information Table S1). Partitioning, thermocycling, and fluorescence acquisition (imaging) were performed according to the manufacturer’s recommendations. Absolute quantification of target and reference genes was obtained using QIAcuity Suite software (Qiagen), and knockdown efficiency was calculated based on normalized expression (Kir gene copies per EF1α copies) relative to dsGFP controls.

### Assessment of *C. Las* Acquisition by ACP

Prior to experimentation, the spray flow rate of the test compound delivered to newly emerging flush was calibrated using an airbrush applicator (Paasche Airbrush Company, Chicago, IL, USA). The volume of compound applied to each replicate was adjusted according to the relative leaf area. To determine leaf area, branches with young flush were secured on a flat cardboard surface alongside a ruler and photographed 1 day prior to treatment. Leaf boundaries were traced using the polygon selection tool in ImageJ^47^ with the Fiji plugin^48^ and areas were calculated using the ruler as a reference scale. Leaf flush areas on *C. Las*-positive plants ranged between 100 and 192 cm^2^, and spray volumes were adjusted accordingly to achieve the desired VU041 concentration on the foliage.

*C. Las*-positive trees were treated with one of three solutions that were comparable to the concentrations used in EPG studies: a control treatment of 0.01% Tween-80, a solution of VU041 at 36 parts-per-billion (ppb) + 0.01% Tween-80, or a solution of VU041 at 365 ppb + 0.01% Tween-80. These concentrations were compared to a negative control of deionized water containing 0.01% Tween-80. Treated flushes were allowed to dry for 2 h before insects were introduced.

For each treatment replication, 12–15 adult ACP (1–3 day-old) were confined to a treated flushing branch of a *C. Las*-positive Valencia SPB-1–14–19 citrus tree. Each treatment consisted of three to six replications (*N* ≈ 45–90 total psyllids). Adults were enclosed in mesh bags on treated branches and provided an acquisition access period (AAP) of either 48 or 72 h. After the AAP, adult mortality was recorded, dead insects were removed, and surviving ACP were collected into 80% ethanol for subsequent DNA extraction and qPCR analysis to quantify *C. Las* infection.

### DNA Isolation and Detection of *C. Las* Using Quantitative Real-Time PCR

DNA from insect samples was isolated using DNeasy Blood and Tissue (Qiagen, Valencia, CA, USA) following the manufacturer’s instructions. DNA concentrations were measured using a NanoDrop 2000 spectrophotometer (ThermoFisher Scientific Inc., Lafayette, CO). Samples were diluted to 15 ng μL^–1^ of ACP DNA for subsequent qPCR analysis targeting *C. Las*-specific 16S rDNA.

Psyllid extracts were amplified using probe-primer sets targeting internal control sequences from the ACP wingless gene, based on published sequences^59,60^ and listed in Supporting Information Table S1. qPCR assays were performed in duplicate using a QuantStudio 6 Flex Real-Time PCR System (Applied Biosystems, Waltham, MA, USA). Each 25.5 μL reaction consisted of 12.5 μL PerfeCTaq PCR ToughMix, 3 μL of each primer, 3 μL of each probe, and 1 μL of DNA template (15 ng). Final primer concentrations were 0.2 μM for both *C. Las* and wingless targets, and final probe concentrations were 0.1 μM. for *C. Las*. Thermal cycling conditions included an initial denaturation at 95 °C for 10 min, followed by 40 cycles of 95 °C for 15 s and 60 °C for 60 s. Each plate containing insect samples included a no template control (NTC), a positive control (*C. Las*-positive DNA extract), and a negative control (*C. Las*-negative DNA extract). Reactions were considered positive for either target if the cycle quantification (CT) value, was ≤36.^4^

### Data Analysis

For topical toxicity assays, concentration–response curves (CRC) were generated using nonlinear regression with a four-parameter logistic (variable slope) model in GraphPad Prism 9 (GraphPad Software, San Diego, California USA). For the VU041-analog toxicity assay data, percentage mortality data were arcsine square-root transformed prior to analysis by one-way analysis of variance (ANOVA) with Tukey’s multiple comparisons test.

Feeding behavior data (xylem and phloem feeding) and acquisition assay data sets were tested for normality using the Kolmogorov–Smirnov test in Prism 9. Percentages related to psyllid feeding behaviors were arcsine square-root transformed and analyzed by ordinary one-way ANOVA with Tukey’s multiple comparisons test. Prior to these analyses, the Brown–Forsythe test was applied to evaluate homogeneity of variances. Data sets with unequal standard deviations (SDs) were analyzed with Welch’s ANOVA followed by Dunnett’s T3 multiple comparisons test, whereas data sets with equal SDs were analyzed using ordinary one-way ANOVA followed by Tukey’s multiple comparisons test. All analyses were conducted in GraphPad Prism 9.

Salivary sheath lengths were averaged across 25 individuals per treatment for three replicates and analyzed using Welch’s ANOVA with Dunnett’s T3 multiple comparisons test (GraphPad Prism version 9.3.1; GraphPad Software, San Diego, CA, USA). Salivary sheath and feeding behavior followed GFP and Kir dsRNA oral delivery were also tested for normality using the Kolmogorov–Smirnov test. Percentage-based feeding behavior data were arcsine square-root transformed and compared using an unpaired *t*-test with Welch’s correction in GraphPad Prism 9.

To assess the statistical significance of knockdown efficiency, the normalized expression (Kir gene copies per EF1α copies) relative to dsGFP controls were compared for each of the three Kir genes. Data sets used were tested for normality using the Kolmogorov–Smirnov test using GraphPad Prism (GraphPad Software). Normalized expression data sets were not normally distributed, therefore a Mann–Whitney *U* test was used to compare normalized expression of the three Kir genes between Kir dsRNA-fed and GDP dsRNA-fed replicates.

## Results

### Toxicity of VU041 to ACP via Topical and Tarsal Contact (Leaf) Exposure

Topical application of VU041 at doses up to 300 ng/insect did not produce measurable toxicity in ACP. However, the addition of 0.01% Tween-80 markedly enhanced topical activity, yielding a 24 h LD_50_ of 25 ng/insect (95% CI: 14–38; Hillslope: 1.2; *r* ^2^: 0.86). A similar pattern was observed for tarsal-contact exposure following foliar application onto newly emerged citrus leaves. No significant mortality was observed at 5 μg/cm^2^ in the absence of Tween-80, but inclusion of 0.01% Tween-80 improved tarsal contact activity of VU041 when applied to citrus leaves. Although no knockdown or toxicity was observed at 1-, 4-, or 6- h postexposure regardless of whether Tween-80 was included, signs of intoxication were observed in >50% of ACP at 24 h, with approximately 50% moribund or dead at 48 h (Figure 1B).

**Figure 1:**
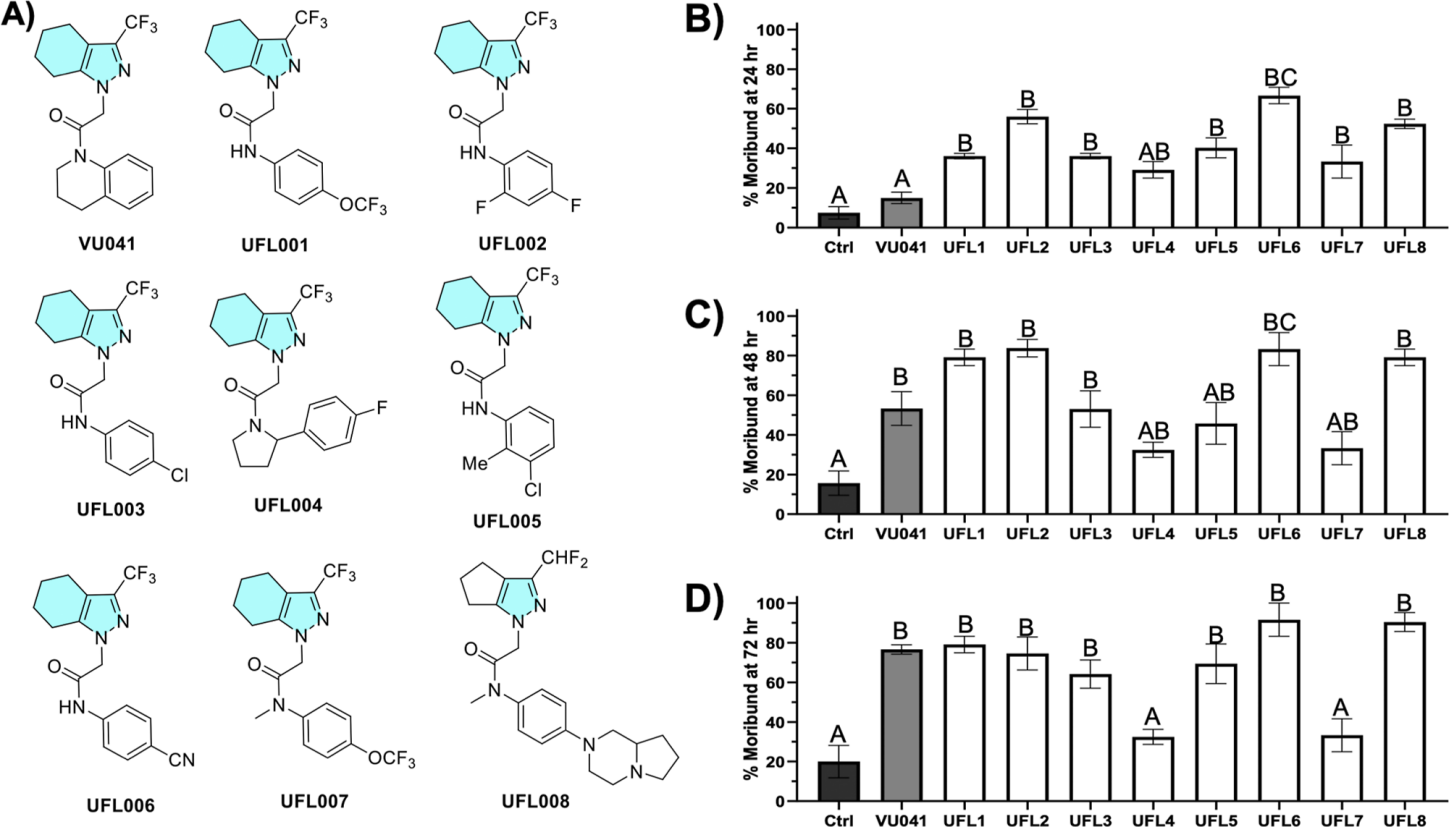
Toxicity of VU041 and structural analogs to adult ACP via tarsal contact exposure on treated leaves. (A) Chemical structures and identifiers of VU041 and VU041 structural analogs. Concentration–response mortality at 10 ng/cm^2^ assessed at 24 h (B), 48 h (C), and 72 h (D) postexposure. All data points represent mean percent mortality ±SEM from three independent biological replicates (*n* = 3). Different uppercase letters above bars indicate significant differences (*P* < 0.05) determined by one-way ANOVA followed by Tukey’s posthoc multiple comparisons test. In panels B–D, % efficacy is defined as ACP that display significant signs of intoxication, unresponsive to stimuli, or dead.

### Toxicity of VU041structural Analogs Assessed by Single-Dose Leaf Dip Bioassay

Substituting the quinoline core of VU041 with para-substituted anilines (UFL001–003, 005–007) produced varied toxicity profiles (Figure 1B). Para-substituted analogs generally exhibited greater toxicity, with the trifluoromethoxy (UFL001) and cyano (UFL006) derivatives showing the highest efficacy across all time points (Figure 1B–D). N-methylation of the aniline nitrogen tended to reduce toxicity (e.g., UFL001 vs UFL007), although UFL008, the N-methylated analog containing bulkier para groups, still showed appreciable activity (Figure 1B–D).

Although the limited number of analogs restricts definitive structure–activity relationship (SAR) conclusions, preliminary analysis revealed consistent associations between physicochemical properties and ACP toxicity independent of time point (Figure 2, Supporting Information Table S2). Predicted lipophilicity (*c*Log*P*) and aqueous solubility (Log*S*) were notably correlated with efficacy (Figure 2B,C). Toxicity showed a negative correlation with lipophilicity (Log*P*, *r* ^2^ = −0.36) (Figure 2A,B) and a positive correlation with aqueous solubility (Log*S*, *r* ^2^ = 0.55) and topological polar surface area (tPSA) (Figure 2C,D). Compounds with higher solubility and greater polarity generally exhibited higher toxicity, with UFL006 demonstrating the greatest efficacy and possessing the highest tPSA value (68.49) (Figure 2D).

**Figure 2:**
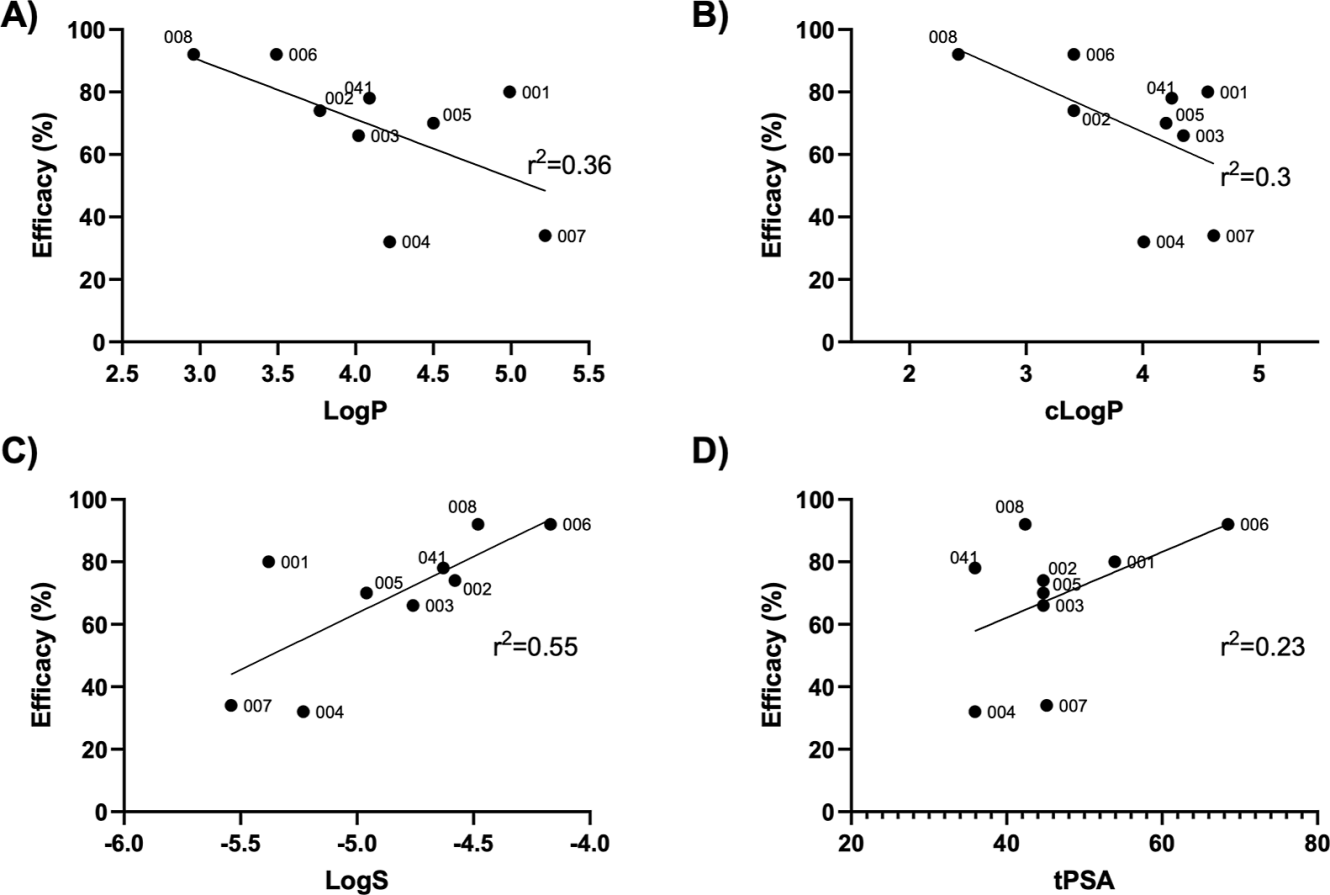
Relationship between physiochemical properties and toxicological efficacy of VU041 analogs. Scatter plots correlating compound physiochemical properties of VU041 and analogs with percent efficacy over 72 h postexposure in adult ACP using single-dose leaf dip bioassay. (A) Lipophilicity (Log*P*) vs efficacy (*r* ^2^ = −0.36) show a moderate negative correlation, indicating that increased lipophilicity reduces compound efficacy. (B) Calculated Log*P* (*C*log*P*) versus efficacy (*r* ^2^ = −0.30) reinforces the negative lipophilicity trend, demonstrating that computational and experimental lipophilicity assessments yield consistent conclusions: more lipophilic compounds are generally less effective against adult ACP. (C) Aqueous solubility (Log*S*) versus efficacy (*r* ^2^ = 0.55) demonstrates the strongest positive correlation among all properties tested, revealing that increased water solubility directly enhances toxicological potency. (D) Topological polar surface area (tPSA) versus efficacy (*r* ^2^ = 0.23) exhibits a moderate positive correlation with molecular polarity, with the most toxic compound (UFL006; tPSA = 68.49) displaying the highest polar surface area. Collectively, these results suggest that optimal efficacy is achieved with compounds possessing high aqueous solubility and greater molecular polarity while minimizing lipophilicity.

Collectively, these results indicate that aqueous solubility and molecular polarity may be critical determinants of activity against ACP for this series of Kir channel inhibitors (Figure 2). Future SAR efforts will therefore emphasize analog designs that increase solubility (higher Log*S*) rather than lipophilicity (lower Log*P*). For the present study, VU041 was selected over the other analogs (Figure 1), for characterization of antifeedant effects due to its synthetic accessibility and the broader opportunities it provides for further structural optimization.

### VU041 Reduces Salivary Sheath Secretion in ACP

The effect of VU041 on ACP gel saliva secretion was assessed by measuring salivary sheath length after 24 h of exposure. Salivary sheaths are feeding structures produced during probing and are essential for successful cell sampling and phloem ingestion and therefore, reductions in sheath length indicate decreased secretory activity of the salivary glands following Kir channel inhibition. Psyllids fed on the control diet produced salivary sheaths with a mean length of 100.4 ± 13.2 μm (Figure 3A). Exposure to VU041 significantly shortened salivary sheaths (Figure 3A), with concentrations of 1 ng, 10 ng, and 100 ng reducing sheath length by approximately 2-fold compared to controls. Representative images of salivary sheaths generated by ACP feeding on 15% sucrose (Figure 3B) versus 100 ng VU041 + 15% sucrose (Figure 3C) illustrate this reduction. Minimal mortality was observed across all treatments during the 24 h feeding assay.

**Figure 3:**
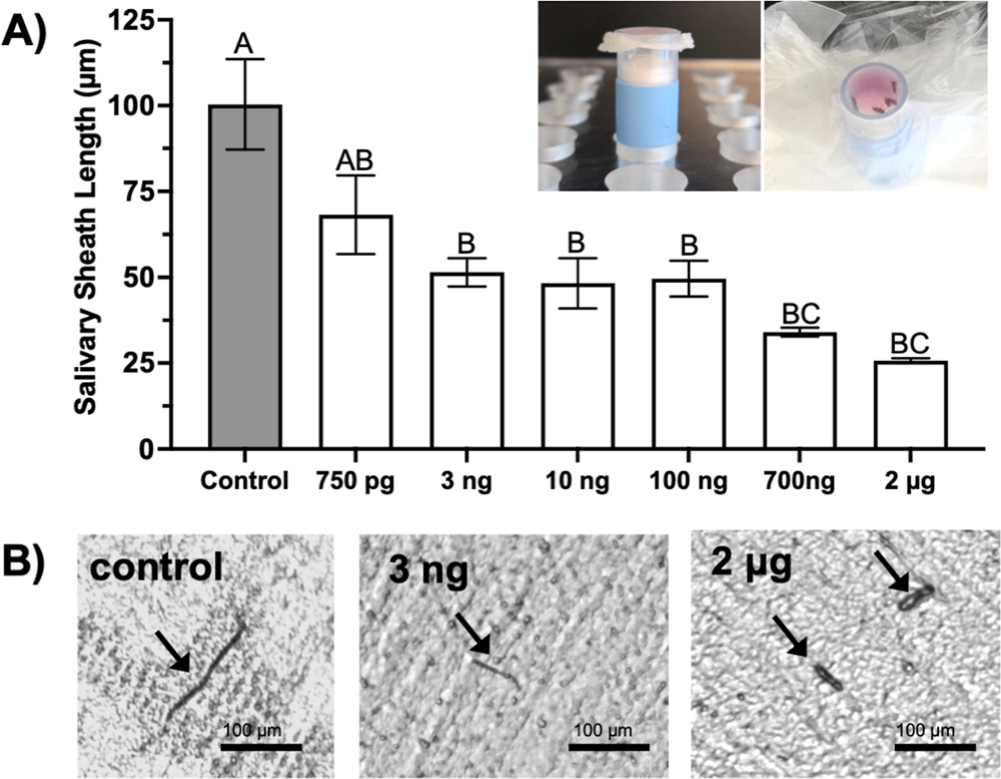
VU041 concentration-dependent inhibition of salivary sheath formation in adult ACP. (A) Salivary sheath length following exposure to increasing VU041 concentrations compared to sucrose-only control (gray bar) in artificial feeding chambers. Mean sheath length ±SEM is shown for *n* = 30 individual ACP per treatment. Different letters above bars indicate statistically significant differences (*P* < 0.05) determined by one-way ANOVA with Tukey’s posthoc test. (B) Representative images of salivary sheaths produced by adult ACP feeding on sucrose solution (control) versus VU041-treated sucrose solutions demonstrating the concentration-dependent reductions in sheath length. Scale bars = 100 μm. Black arrows in panes of panel B indicate salivary sheaths.

### Foliar Applications of VU041 Prevent Phloem and Xylem Feeding by ACP Adults

Given that VU041 reduced salivary gland secretory activity, we next quantified the effects on feeding behavior using EPG recordings on citrus leaves. Representative traces for control and VU041-treated leaves are shown in Figure 4. ACP feeding on control leaves (water treatment +0.01% Tween-80) exhibited the expected and previously documented probing patterns, including C waveforms (intercellular apoplastic stylet pathway), D waveforms (initial contact with phloem sieve elements), E1 waveforms (salivation into sieve elements), E2 waveforms (phloem sap ingestion), and G waveforms (active xylem uptake) (Figure 4A).

**Figure 4:**
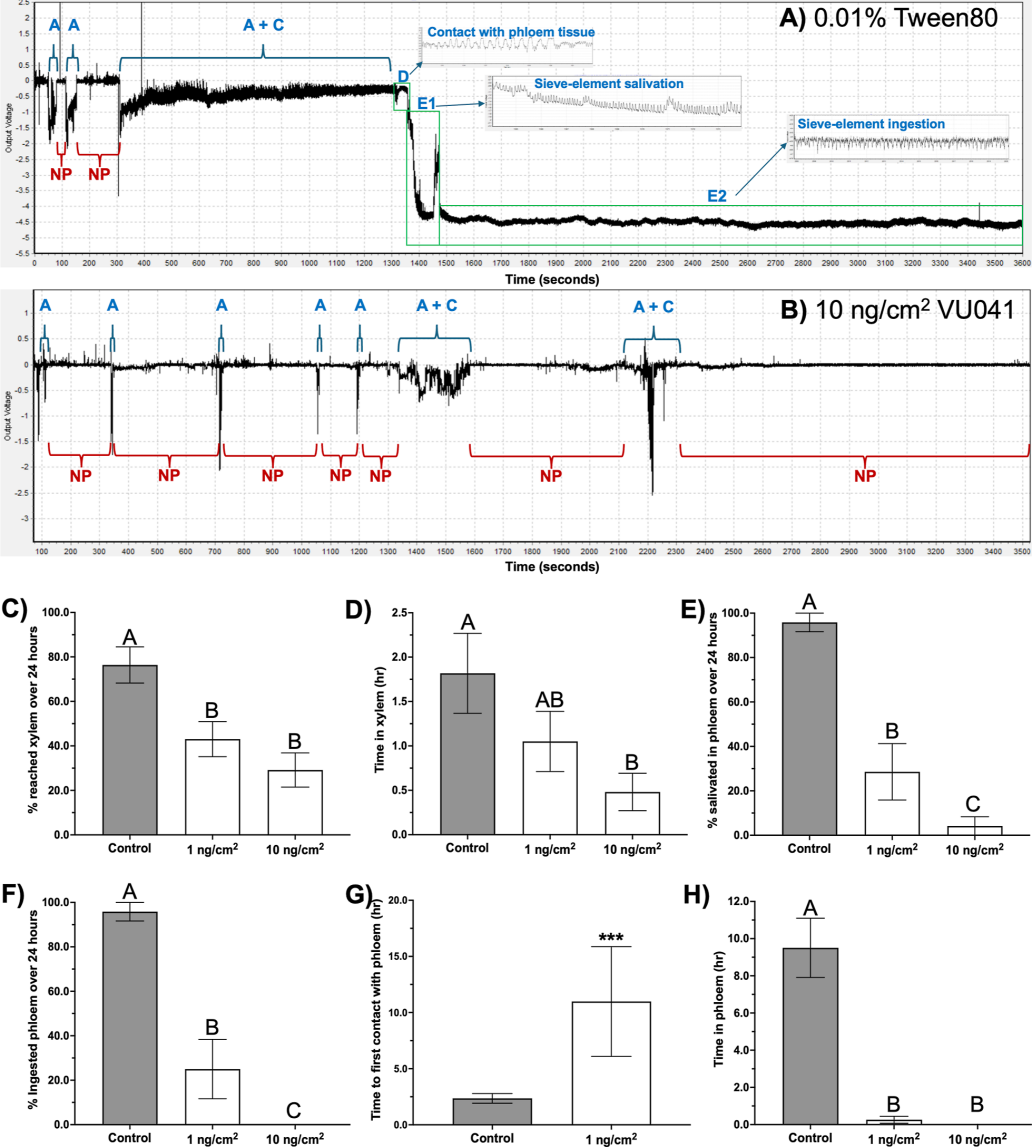
VU041 disrupts phloem feeding and salivation behaviors in adult ACP. (A) Representative electrical penetration graph (EPG) recording from control ACP showing normal probing behavior: initial probe initiation (A-waveform), nonprobing periods (NP), stylet pathway activity with gelling saliva secretion (C-waveform), first phloem tissue contact (D-waveform), salivation into phloem sieve elements (E1-waveform; associated with *C. Las* inoculation), and phloem sap ingestion (E2-waveform; associated with *C. Las* acquisition). (B) Representative EPG recording from an adult ACP on a VU041-treated leaf demonstrating disrupted probing, prolonged nonprobing periods, and failure to reach, salivate in, or ingest from phloem (notably absent D, E1, and E2 waveforms). (C–H) Quantitative EPG-derived probing and feeding behaviors recorded over 24 h for adult ACP placed on the adaxial surface of young citrus leaves treated with control or VU041 solutions, including: (C) percentage of ACP reaching and ingesting from xylem (G-waveform), (D) total duration of xylem feeding (G-waveform), (E) percentage of ACP reaching and salivating in phloem (D + E1 waveforms), (F) percentage of ACP ingesting phloem sap (E2-waveform), (G) latency to first phloem contact (D-waveform), and (H) total duration of phloem feeding (D + E1 + E2 waveforms). All bars represent mean values ±SEM. Statistical significance was determined by one-way ANOVA with Tukey’s posthoc multiple comparisons or Welch’s ANOVA (for unequal variances) with Dunnett’s T3 posthoc test; bars not sharing the same letter differ significantly (*P* < 0.05). In panel G, asterisks indicate statistical significance (*P* < 0.001) determined by unpaired *t*-test.

In contrast, ACP feeding on leaves treated with VU041 displayed noticeably disrupted probing, shorter feeding bouts, and prolonged nonprobing (NP) intervals (Figure 4B). Foliar application of VU041 to young Valencia SPB-1–14–19 leaves significantly reduced xylem feeding, with 1 ng/cm^2^ and 10 ng/cm^2^ treatments lowering the proportion of psyllids that fed from xylem by 1.8-fold and 2.6-fold, respectively (Figure 4C). A 3.7-fold reduction in total xylem feeding time was also observed at 10 ng/cm^2^ (Figure 4D; *P* < 0.05).

VU041 strongly inhibited phloem feeding at 10 ng/cm^2^ with only a single E1 salivation event recorded across all ACP (*n* = 24), and no E2 waveforms were observed during the 24 h recording period (Figure 4E,F). At 1 ng/cm^2^, VU041 also significantly reduced the percentage of psyllids exhibiting E1 and E2 waveforms by 3.4-fold and 3.8-fold, respectively, when compared to controls (Figure 4E,F). No significant differences in phloem feeding were detected between the 1 ng/cm^2^ and 10 ng/cm^2^ treatments (Figure 4E,F).

Exposure to VU041 at 1 ng/cm^2^ significantly increased the time required for psyllids to reach phloem tissues (time to first D-waveform; *P* < 0.0001; Figure 4G). Statistical comparisons were not possible for the 10 ng/cm^2^ treatment because only one of 24 ACP individuals reached the phloem, and only for 2.4 min (Figure 4G,H). Overall, foliar application of VU041 at 1 ng/cm^2^ reduced phloem feeding duration by 8-fold relative to controls, representing a statistically significant reduction (*P* < 0.0001; Figure 4H).

### Knockdown of ACP Kir Genes Reduces Salivary Gland Secretion and Phloem Feeding

To determine whether the pronounced changes in ACP feeding behavior and *C. Las* acquisition (described below) observed after VU041 treatment were due to Kir channel inhibition rather than off target effects, adult ACP were fed a mixture of Kir1, Kir2A, and Kir2B dsRNAs for 120 h, after which feeding behaviors were quantified. Digital PCR quantification revealed differential dsRNA-mediated silencing among Kir gene family members. Kir1 expression in dsKir-treated psyllids was reduced by 49.1% relative to dsGFP controls (*p* = 0.0015; Figure 5A). Kir2A expression was also significantly reduced, although to a lesser extent of 27.7% (*p* = 0.016) (Figure 5A). In contrast, Kir2B transcripts were not significantly affected by dsKir treatment (2.6%; *p* = 0.542) (Figure 5A). These results indicate effective silencing of Kir1 and Kir2A, but not Kir2B, under the conditions tested.

ACP fed Kir dsRNAs produced significantly shorter salivary sheaths than those fed GFP dsRNA controls (*P* < 0.01) (Figure 5B). In addition, psyllids fed on Kir dsRNA in 25% sucrose for 120 h reached the phloem, salivated, and ingested phloem sap approximately 4-fold less frequently than GFP dsRNA-fed controls (*P* < 0.001; Figure 5C,D).

### Foliar Application of VU041 Induces Mortality and Reduces *C. Las* Acquisition in ACP Adults

The substantial impacts of VU041 on ACP survival and feeding prompted us to evaluate whether foliar treatments would also reduce bacterial load and the proportion of psyllids acquiring *C. Las*. To test this, a caged acquisition assay was conducted using newly emerged leaf flushes from *C. Las*-positive trees treated with 36 or 365 ppb VU041, with acquisition access periods (AAP) of either 48 h (Figure 6A–C) or 72 h (Figure 6D–F). At 48 h, VU041 significantly increased ACP mortality by 2-fold and 6.3-fold at 36 and 365 ppb, respectively, compared with controls (Figure 6A). The highest mortality (average of 46 ± 3%) occurred at 365 ppb (Figure 6A). Foliar application also reduced *C. Las* titers in surviving psyllids by 1.7-fold and 3-fold at 36 and 365 ppb, respectively (*P* < 0.001; Figure 6B). Additionally, treatment with 365 ppb VU041 resulted in a 3.6-fold decrease (*P* < 0.01) in the proportion of ACP testing positive for *C. Las* compared with controls (Figure 6C).

At the 72 h AAP, evaluation of the 365 ppb treatment was not possible because mortality reached 96 ± 2% (Figure 6D). However, at 36 ppb, mortality averaged 27 ± 1% at 72 h, and surviving psyllids exhibited a 2-fold reduction (*P* < 0.001) in *C. Las* abundance relative to controls (Figure 6D,E). Although the percentage of surviving ACP that tested positive for *C. Las* at 36 ppb did not differ statistically from controls at 72 h, these values do not account for individuals that died during the assay (Figure 6F).

**Figure 5:**
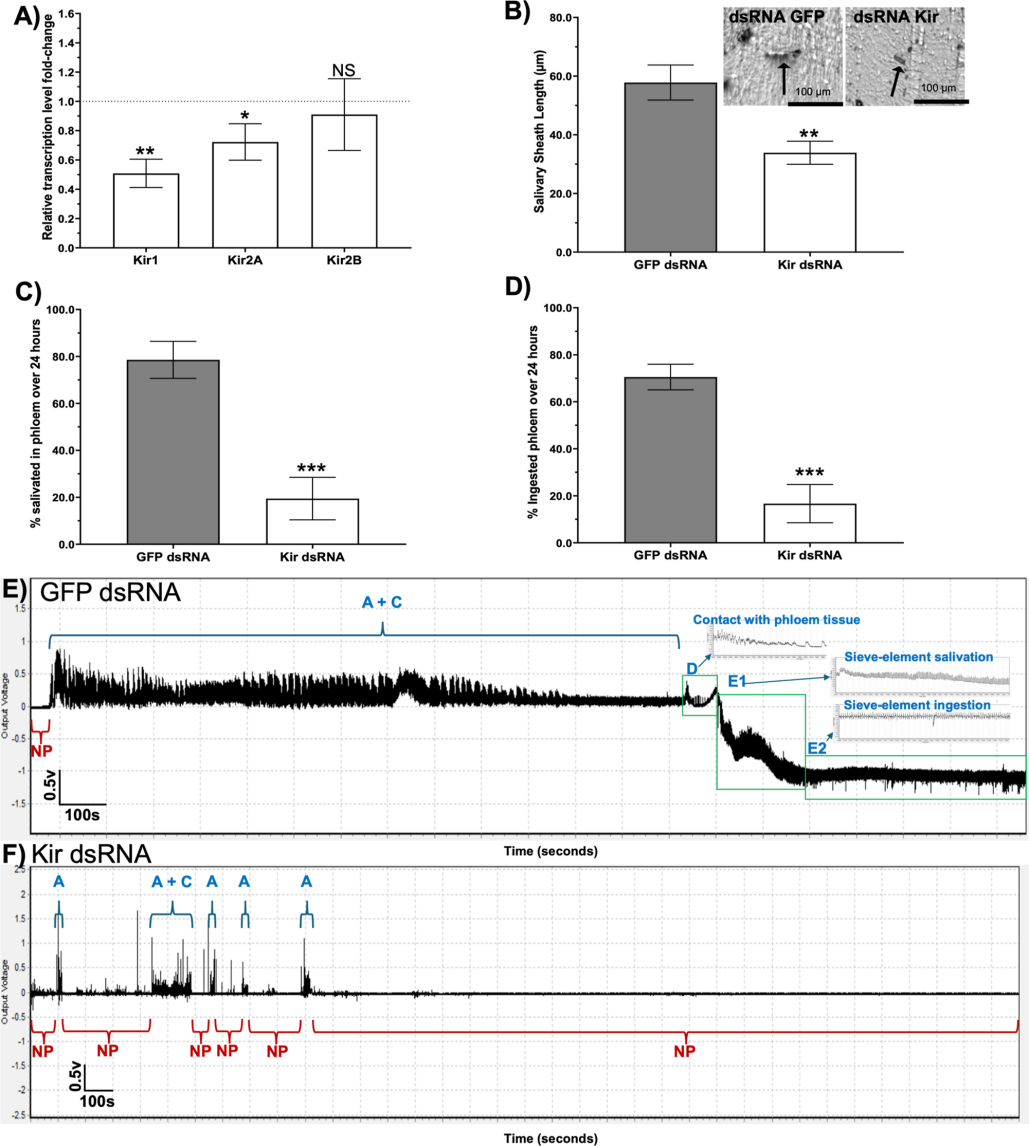
Kir gene knockdown reduces salivation and phloem feeding in adult ACP. (A) Quantification of mRNA knockdown for Kir1, Kir2A, and Kir2B transcripts measured 120 h post-dsRNA ingestion, demonstrating successful gene silencing related to GFP control treatment. (B) Salivary sheath length measured from salivary glands dissected from Kir knockdown and GFP control adult ACP, showing reducing gel-saliva secretion following Kir dsRNA treatment. Mean salivary sheath length ±SEM is presented for *n* = 30 individuals per treatment group. Inset images (panel B) show representative salivary sheath lengths from GFP control (left) and Kir knockdown (right) ACP; scale bars in insets represent 100 μm. Black arrows indicate salivary sheath. (C,D) Quantitative feeding behaviors measured over 24 h via EPG analysis of adult ACP pretreated with Kir dsRNA or GFP control for 5 days prior to placement on the adaxial surface of young citrus leaves. Metrics include: (C) percentage of ACP reaching and salivating in phloem (D + E1 waveforms) and (D) percentage of ACP ingesting phloem sap (E2 waveform). All bars represent means ± SEM. Different letters above bars indicate statistically significant different (*P* < 0.05) determined by one-way ANOVA with Tukey’s posthoc multiple comparisons test or Welch’s ANOVA (for unequal variances) with Dunnett’s T3 posthoc test. (E,F) Representative EPG traces of GFP dsRNA (E) and Kir dsRNA (F). Behavior described are initial probe initiation (A-waveform), nonprobing periods (NP), stylet pathway activity with gelling saliva secretion (C-waveform), first phloem tissue contact (D-waveform), salivation into phloem sieve elements (E1-waveform; associated with *C. Las* inoculation), and phloem sap ingestion (E2-waveform; associated with *C. Las* acquisition).

## Discussion

Managing ACP populations remains essential for preventing HLB occurrences and restoring citrus productivity, with chemical control still the most effective strategy available.^15^ Considering this, our goal was to develop commercially viable chemical tools that reduce HLB through two complementary mechanisms: (1) inducing acute toxicity at field-relevant concentrations and (2) suppressing salivary gland activity to inhibit plant feeding at sublethal concentrations. This dual-action approach aligns with grower needs for insecticides that rapidly kill ACP while also offering an additional protective benefit—preventing feeding and salivation on treated trees.

The Kir inhibitor, VU041,^39^ was toxic to ACP at low nanogram doses/concentrations following topical or contact (leaf) exposure (Figure 1B,C) that was comparable to activity of commercial insecticides currently labeled for ACP control.^43^ Importantly, VU041 is significantly (ca. 1000-fold) more toxic to ACP than flonicamid^61^ which is a nanomolar inhibitor of hemipteran Kir channels.^62^ In addition to toxicity, the rate of intoxication of VU041 was significantly greater than flonicamid with VU041 inducing mortality to ACP between 24 and 48 h and flonicamid inducing mortality at 7 days post exposure.^61^

Interestingly, VU041 was only toxic when 0.01% Tween-80 was included in the treatment solution regardless of topical or leaf contact exposure methods, which is similar to that observed with flonicamid.^61^ Although VU041 combined with an adjuvant resulted in acute (24 h) toxicity via topical application, the inclusion of Tween-80 did not result in 24 h toxicity when applied onto the citrus leaves with approximately 50% showing signs of intoxication at 24 h and significant mortality not being observed until 48 h (Figure 1B,C). These data indicate poor delivery of VU041 to ACP after leaf treatments even with the addition of Tween-80 as an adjuvant. Optimizing bioavailability is especially important in citrus systems due to their relatively thick leaves and substantial wax layers, both of which can bind lipophilic insecticides and limit their uptake by insects.^63–65^ Nonionic surfactants, such as Tween-80, are commonly incorporated into agrochemical formulations to enhance the bioavailability of active ingredients by improving droplet spreading, deposition, and retention on insect cuticle or plant surfaces.^66^ For example, these surfactants reduce solution surface tension, increasing the wetted area and prompting more uniform distribution of pesticide droplets on waxy surfaces.^66^ Improved droplet spread across the leaf surface area versus absorption within the waxy epicuticle of the citrus leaf is likely to increase bioavailability and potentially, improve penetration of VU041 through the ACP cuticle to improve VU041-mediated toxicity. Given the >100-fold increase in VU041 toxicity observed on citrus leaves with Tween-80 versus without addition of the adjuvant, future work should prioritize identifying optimal adjuvants that maximize cuticular penetration of VU041 and related analogs after leaf treatments.

The high toxicity of VU041 to ACP, combined with its lack of acute toxicity to honey bees,^39^ suggests that this class of molecules has strong potential for incorporation into ACP management programs. However, the acute toxicity observed in ACP was somewhat unexpected, as previous studies reported that Kir inhibitors did not cause acute mortality in aphids, with significant mortality only occurring after 96 h and attributed to feeding inhibition.^18,38^ Notably, earlier toxicity studies in mosquitos and aphids did not include Tween-80 or other adjuvants, which may partly explain the lower toxicity reported for these species.

The pronounced acute toxicity of VU041 to ACP raises important questions about its mode of action, which is unlikely to be explained solely by salivary gland inhibition or feeding cessation. Recent work indicates that Kir channels are expressed in insect neural tissue, where they play key roles in potassium spatial buffering mediated by astrocyte-like glia,^67^ contribute to synaptic transmission at the neuromuscular junction,^26^ and modulate GABAergic signaling through functional coupling with potassium-chloride cotransporters.^33,68^ Although additional research is required to elucidate the precise mechanisms underlying VU041-mediated mortality in ACP, it is plausible that interference with ACP Kir channel function disrupts neural processes in ways that critically impair nervous system function.

Beyond their role in acute lethality, Kir channels represent a promising molecular target for inducing salivary gland dysfunction and suppressing feeding behavior through antifeedant activity in both hematophagous and sap-feeding arthropods.^19,35–37,69^ While Kir1 and Kir2 expression in ACP salivary gland was less than that of the Malpighian tubules or midgut tissues,^61^ Kir channel mRNA is highly expressed in the salivary glands of the brown planthopper (*Nilaparvata lugens*) and the common fruit fly (*Drosophila melanogaster* L.), where it is linked to regulation of salivary secretory activity.^29,70,71^ For example, chemical inhibition of Kir channels or salivary gland-specific RNAi knockdown in *D. melanogaster* reduces sucrose ingestion;^29^ Kir1 inhibition also decreases salivary secretion and feeding behavior in *N. lugens* ^70,72^ whereas Kir inhibition with VU041 reduces gel saliva production and prevents phloem feeding by aphids on cotton.^18^

These findings motivated us to examine whether VU041 similarly reduces salivary gland secretory activity in ACP and whether foliar application inhibits phloem feeding on citrus leaves. Indeed, oral exposure to VU041 significantly reduced gel saliva secretion in individual ACP, which corresponded to strong inhibition of phloem feeding on VU041-treated citrus foliage (Figures 3–4). Importantly, these results were corroborated by RNAi-mediated knockdown of Kir channel genes, with a 49% reduction of Kir1 expression and a 27.7% reduction of Kir2A expression leading to significant decreases in salivary gland activity and phloem feeding (Figure 5). These knockdown data validate Kir channels as the physiological regulators of ACP salivary secretion and phloem feeding and in turn, strengthening the conclusion that the antifeedant effects of VU041 are due to Kir inhibition rather than off-target mechanisms.

It is noteworthy that the ratio of watery and gel saliva in aphids is influenced by chemosensory and mechanosensory feedback detected by sensilla in the precibarium and on the stylet tip.^73,74^ This raises the possibility that ACP Kir channels may also modulate these sensory structures, thereby altering salivary composition and stylet navigation, which is an alternative or complementary mechanism to the current hypothesis that salivary gland Kir channels primarily maintain ionic homeostasis across secretory cells.^29^ Future work is needed to elucidate the precise role of ACP Kir channels in salivary gland physiology and sensory sensilla function as well as the mechanistic linkage between Kir channels, saliva secretion, and phloem feeding behavior.

From a disease-management perspective, the reduction in *C. Las* acquisition observed in VU041-exposed survivors is expected to translate directly into improved tree health and yield. HLB transmission is governed jointly by the abundance of ACP and the proportion of the population that is infectious. Quantitative transmission studies demonstrate that even low inoculation probabilities per individual psyllids can scale to high infection risk when many psyllids acquire *C. Las*, whereas reducing the fraction of infected vectors sharply decreases tree-level infection probability. For example, Pelz-Stelinski et al. (2010) showed that individual adults transmit *C. Las* at only 4–10%, but groups of ≥100 psyllids transmit at rates approaching 90%, illustrating the nonlinear effect of infected vector numbers on disease spread.^4^ Similarly, Ammar et al. (2016) found that psyllids acquiring *C. Las* as nymphs develop higher bacterial titers and are more likely to inoculate citrus than adults acquiring the pathogen later, indicating that any treatment that suppresses acquisition during feeding stages reduces the pool of competent vectors.^3^ At the groove scale, Lee et al. (2015) used microsimulation modeling to show that HLB epidemics are propelled by repeated cycles of vector acquisition and reinoculation; without control, grooves can reach ∼12,000 psyllids per tree within a year, whereas reducing psyllid populations by ∼75% during flush dramatically lowers the proportion of trees with high infection pressure.^75^ Our finding that VU041 produces both high psyllid mortality and reduced *C. Las* acquisition in survivors therefore indicates a compounded epidemiological benefit by decreasing both vector density and the proportion of infectious individuals. This conclusion is reinforced by field studies using trunk-injected oxytetracycline, in which reduced *C. Las* titers within trees were associated with lower acquisition and transmission by ACP and concurrent improvements in canopy vigor, fruit yield, and juice quality.^76^ Moreover, long-term economic analyses in Florida demonstrate that more intensive suppression of psyllid populations is consistently associated with higher fruit yield and reduced HLB severity,^77^ showing that continued reduction of vector numbers and transmission potential benefits citrus productivity even in highly infected grooves. Together, these empirical and modeling results support the expectation that Kir-targeting chemistries such as VU041, by combining strong toxicity with suppressed pathogen acquisition, should confer additive benefits for HLB management at the groove scale.

**Figure 6:**
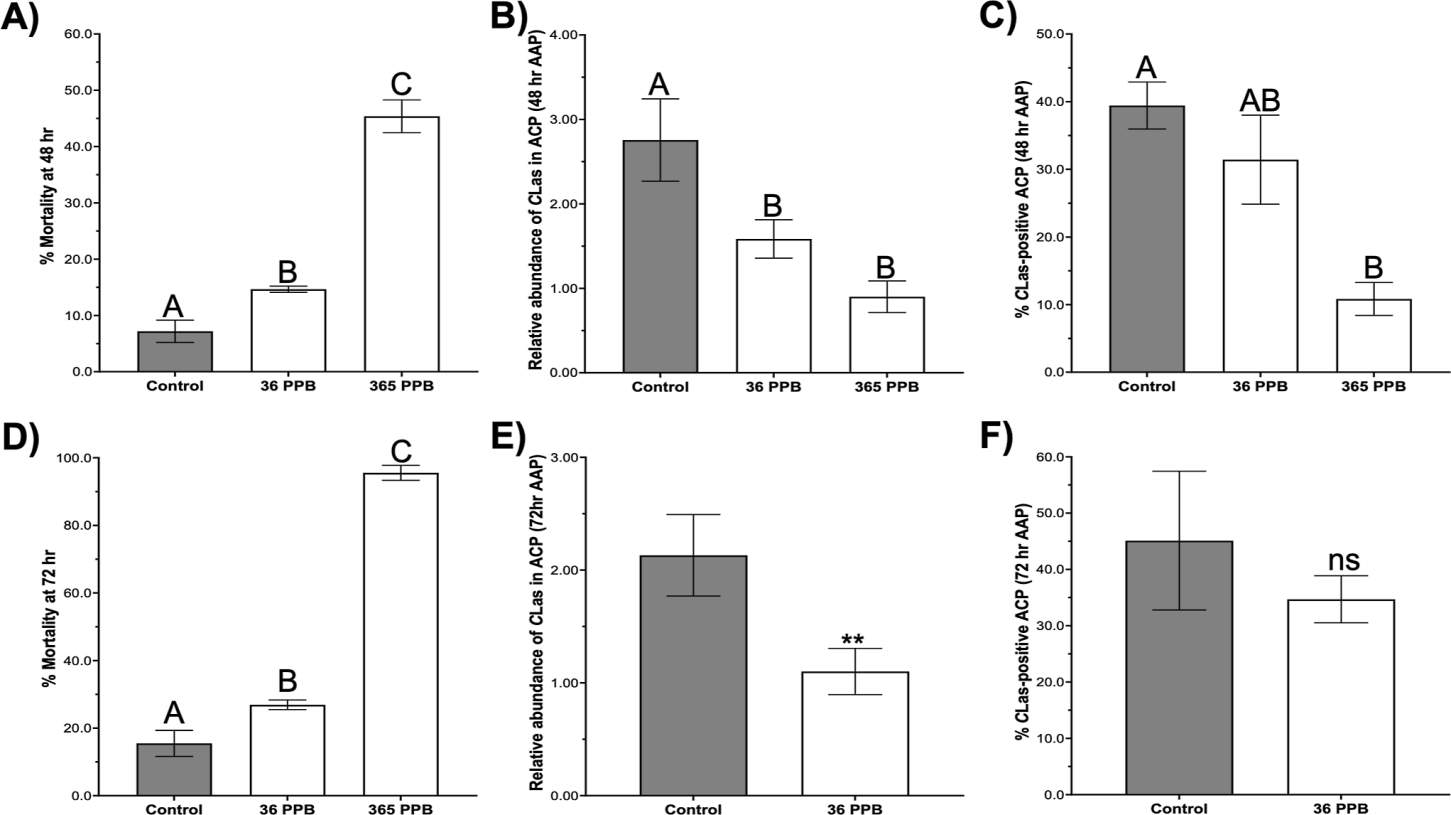
VU041 foliar treatment reduces ACP survival and *C. Las* acquisition on citrus. Caged acquisition assays were conducted on new flushes of *C. Las*-positive citrus trees treated with VU041 at 1 ng/cm^2^ or 10 ng/cm^2^, with acquisition access periods (AAPs) of 48 h (A–C) or 72 h (D–F). (A) Percent mortality of *C. Las*-exposed ACP following a 48 h AAP on VU041-treated foliage. (B) Relative abundance of *C. Las* in surviving ACP collected after the 48 h AAP, quantified by qPCR. (C) Percentage of ACP testing positive for *C. Las* (infection prevalence) after the 48 h AAP. (D) Percent mortality of *C. Las*-exposed ACP following a 72 h AAP on VU041-treated foliage. (E) Relative abundance of *C. Las* in surviving ACP collected after the 72 h AAP, quantified by qPCR. F) Percentage of ACP testing positive (infection prevalence) for *C. Las* after the 72 h AAP. All bars represent mean values ±SEM. Different letters above bars indicate statistically significant differences (*P* < 0.05) determined by one-way ANOVA with Tukey’s posthoc multiple comparisons test.

Intensive management of ACP with insecticides has causes widespread evolution of resistance throughout Florida and in other countries, including China, Mexico, and Brazil with reported decreases in sensitivity reaching up to 4000-fold.^78^ In response, we sought to develop a strategy incorporating two complementary mechanisms to prevent *C. Las* transmission. We report the discovery that VU041, which is an established Kir-directed insecticide,^32,39^ is acutely toxic to ACP, producing rapid knockdown. Acute toxicity is a highly desirable outcome for growers, as this mode of vector control directly reduces disease pressure and requires minimal extension or outreach for adoption. In addition, VU041 inhibited phloem feeding at sublethal concentrations, providing a secondary mechanism to prevent *C. Las* transmission even when acute lethality is diminished, which may be due to variable foliar coverage. Unlike many antifeedants that exhibit a latency period,^49^ VU041 acts rapidly and prevents ACP from initiating phloem feeding shortly after exposure. Importantly, *C. Las* acquisition was significantly reduced in psyllids that survived an LC_50_ exposure, and when combined with high mortality, this dual action is likely to enhance citrus tree health and yield.^79,80^ The combined toxicity, antifeedant activity, and reduced acquisition of *C. Las* observed following VU041 exposure indicate that this chemical scaffold has strong potential for HLB prevention and represents a promising lead for developing tools to mitigate the severe citrus production losses caused by HLB.

## Supplementary Material



## Notes

Supporting Information contains two tables that list the primers used to detect *D. citri* housekeeping genes and *C. Las* (Suppl Table S1) and the physiochemical properties of VU041 analogs (Table S2) (PDF)
