# Supplementary material for: Inward Rectifier Potassium (Kir) Channel Inhibitors Protect Citrus from the Asian Citrus Psyllid by Inducing Toxicity and Inhibition of Feeding
Source: J Agric Food Chem. 2026 Mar 24;74(13):10892–905. doi: 10.1021/acs.jafc.5c17254 (PMC13067347; doi:10.1021/acs.jafc.5c17254)
Supplement: Supplementary file 1 [file jf5c17254_si_001.pdf]

Supplemental Information for:

Inward rectifier potassium (Kir) channel inhibitors protect citrus from the Asian citrus psyllid by inducing toxicity and inhibition of feeding

Authors: Flinn O'Hara[1], Alexandra Cremades[1], Na Xie[1], Erik L Roldán [2], Miltan Chandra Roy [2], Sandipa Gautam [3], Mamoudou Setamou [4], Amar Chittiboyina[5], Troy D Anderson[6], Lukasz L. Stelinski[2], Daniel R. Swale[1*]

Author Affiliations:

[1] Emerging Pathogens Institute, Department of Entomology and Nematology[2], University of Florida, Gainesville, FL 32610

[2] Citrus Research and Education Center, Department of Entomology and Nematology, University of Florida, Lake Alfred, FL, USA

[3] University of California Agricultural and Natural Resources, Statewide IPM Program, Exeter, CA, USA

[4] Citrus Center, Texas A&M University Kingsville, Weslaco, TX, USA

[5] National Center for Natural Products Research, School of Pharmacy, University of Mississippi, University, MS, 38677, USA

[6] Department of Entomology, University of Nebraska, Lincoln, NE 68583

***Corresponding Author**:

Daniel R. Swale, Ph.D.
Emerging Pathogens Institute
Department of Entomology and Nematology
University of Florida
Gainesville, FL 32610, USA
dswale@ufl.edu

Supplemental Tables

**Supplemental Table 1:** List of primers and probes used to detect *D citri* housekeeping gene (wingless) and *C*Las (16S) using quantitative PCR			
Target species	Target gene	Primer/ probe sequence	References
*C*L*as*	16S rDNA	P -50-TCGAGCGCGTATGCGAATAC-30	[59]
		F-50-GCGTTATCCCGTAGAAAAAGGTAG-30	
		R -50-AGACGGGTGAGTAACGCG-30	
D. citri	wingless	P -50-GCTCTCAAAGATCGGTTTGACGG-30	[60]
		F-50-GCTGCCACGAACGTTACCTTC-30	
		R-50-TTACTGACCATCACTCTGGACGC-30	
P=probes, F= forward primer, and R=reverse primer			

**Supplemental Table 2:** Physiochemical properties of VU041 and respective analogs									
	**VU041**	**UFL001**	**UFL002**	**UFL003**	**UFL004**	**UFL005**	**UFL006**	**UFL007**	**UFL008**
**LogP***	4.09	4.99	3.77	4.02	4.22	4.5	3.49	5.22	2.96
**tPSA***	35.91	53.93	44.7	44.7	35.91	44.7	68.49	45.14	42.39
**CLogP***	4.25	4.56	3.41	4.35	4.01	4.2	3.41	4.61	2.42
**LogS***	-4.63	-5.38	-4.58	-4.76	-5.23	-4.96	-4.17	-5.54	-4.48
**Efficacy**$^{\#}$	78	80	74	66	32	70	92	34	92
* These physicochemical properties were generated using ChemDraw, ver. 25.0.2.14; Only efficacy data post 72 h treatment considered.									
